# Mining the A.E. Watkins Wheat Landrace Collection for Variation in Starch Digestibility Using a New High-Throughput Assay

**DOI:** 10.3390/foods12020266

**Published:** 2023-01-06

**Authors:** Petros Zafeiriou, George M. Savva, Jennifer H. Ahn-Jarvis, Frederick J. Warren, Marianna Pasquariello, Simon Griffiths, David Seung, Brittany A. Hazard

**Affiliations:** 1 Quadram Institute Bioscience, Norwich NR4 7UQ, UK; 2 John Innes Centre, Norwich NR4 7UH, UK

**Keywords:** wheat, landrace, starch digestibility, natural variation, high-throughput, retrograded starch, starch structure, screening tool, pipetting tool, breeding

## Abstract

Breeding for less digestible starch in wheat can improve the health impact of bread and other wheat foods. The application of forward genetic approaches has lately opened opportunities for the discovery of new genes that influence the digestibility of starch, without the burden of detrimental effects on yield or on pasta and bread-making quality. In this study we developed a high-throughput in vitro starch digestibility assay (HTA) for use in forward genetic approaches to screen wheat germplasm. The HTA was validated using standard maize and wheat starches. Using the HTA we measured starch digestibility in hydrothermally processed flour samples and found wide variation among 118 wheat landraces from the A. E. Watkins collection and among eight elite UK varieties (23.5 to 39.9% and 31.2 to 43.5% starch digested after 90 min, respectively). We further investigated starch digestibility in fractions of sieved wholemeal flour and purified starch in a subset of the Watkins lines and elite varieties and found that the matrix properties of flour rather than the intrinsic properties of starch granules conferred lower starch digestibility.

## 1. Introduction

Starchy foods are a main source of carbohydrate in our diet and an important source of energy. Reducing the rate and extent of starch digestibility in foods can help to maintain healthy blood glucose levels, which is important for the prevention and management of obesity and chronic diseases like type II diabetes [1]. Furthermore, there is substantial evidence that consumption of resistant starch, that is the starch that escapes digestion in the small intestine and reaches the colon, can reduce blood glucose and help to maintain a healthy gut [2,3,4]. Wheat (*Triticum aestivum* L.) is one of the most widely consumed crops worldwide and provides up to 50% of the calories in the human diet, mainly in the form of starch, so improving its nutritional quality could deliver health benefits to a large number of people [5,6]. Thus, breeding for wheat with less digestible starch is an important strategy to develop healthier foods to reduce dietary risk factors of chronic diseases.

A mature wheat grain contains predominately starch, 60–70% (*w*/*w*), which is the greatest contributor to calories, and also 10–15% (*w*/*w*) protein and 11–15% (*w*/*w*) dietary fiber [7,8]. Starch is produced in the endosperm of wheat during grain filling and is composed of two distinct α-glucan polymers, amylose (linear α-1,4-linked chains) and amylopectin (α-1,4-linked chains with α-1,6-linked branches). In their native state, starch polymers exist as partially crystalline granules, which are difficult for humans to digest; thus starch-based foods are typically cooked prior to consumption. Heating starch in the presence of water leads to starch gelatinization making starch polymers more digestible [9,10,11]. Subsequent cooling leads to retrogradation in which starch polymers form crystalline structures that are less digestible [12,13]. Moreover, there are several other factors which contribute to differences in starch digestibility such as starch molecular structure [14,15,16] and properties of wheat flour (particle size and protein content) [17,18].

To date, reverse genetic studies in wheat have demonstrated potential for increasing resistant starch levels using induced mutations in starch biosynthesis genes [14,19,20,21]. However, initial analyses of some mutants have shown detrimental effects on yield and on pasta and bread-making quality, so identifying additional sources of genetic variation for starch digestibility could support the development of improved traits for commercial breeding applications [14,20]. Wheat landraces, locally adapted lines that have not been modified through modern breeding techniques, present reservoirs of genetic diversity that can be introduced into modern varieties. Of special note is the A.E. Watkins bread wheat landrace collection, encompassing 826 bread wheat landraces collected in the 1920s and 1930s from a global geographic distribution. This collection showed a greater level of genetic diversity compared to modern elite European bread wheat varieties and has been used to identify resistance genes for a variety of diseases [22,23,24,25,26]. The Watkins lines have been purified by single-seed descent from which many genomic and genetic resources have been developed. A core set of 118 accessions (c.Watkins) capturing most of the genetic diversity in the Watkins collection was presented by Wingen et al. [22], and used to generate nested association mapping populations, all of which were genotyped, have genetic maps available, and are free to access (http://wisplandracepillar.jic.ac.uk/) (accessed on 16 November 2022). Thus, a key aim of this study was to determine the extent of natural variation in starch digestibility of the c.Watkins lines and in modern elite UK varieties (bread, biscuit, and animal feed varieties recommended on the UK Agriculture and Horticulture Development Board Recommended List, AHDB).

Despite the availability of diverse wheat germplasm resources like the c.Watkins collection, forward screening approaches for starch digestibility have been limited due to the lack of informative, accurate, and efficient phenotyping methods. Screening based on amylose content, which has a positive association with resistant starch content, can identify lines with high levels of resistant starch but cannot identify factors beyond amylose content that may cause resistance to digestion [27,28]. Only a few studies have developed methods to directly screen large populations for starch digestibility and these have focused on analyzing purified starch [29]. However, other components of the wheat flour matrix and processing (e.g., starch retrogradation) could potentially impact starch digestibility [30,31,32]. To our knowledge, no previous studies have screened flour samples of wheat germplasm collections.

**Figure 1:**
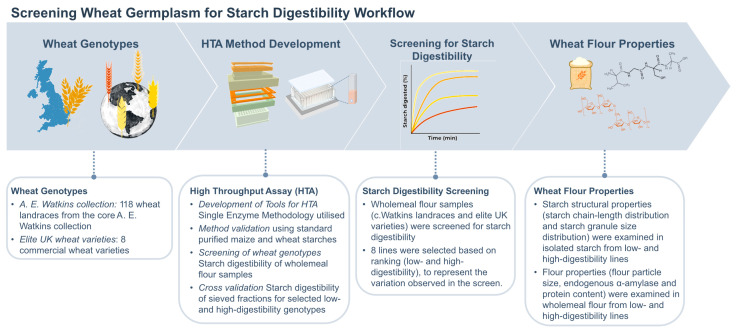
Study workflow for screening starch digestibility of wheat germplasm.

To facilitate rapid assessment of starch digestibility in the c.Watkins wheat landraces, an in vitro single-enzyme system was utilized (Figure 1, study workflow) [30,33,34]. This system has proved useful for measuring starch digestibility in mechanistic studies and early-stage food product development and produces results which are well correlated with human glycaemic responses to foods [33,35]. Moreover, compared to other in vitro digestion methods, the single-enzyme system has advantages such as fewer steps, low consumables, and non-specialized equipment which support its adaptation to a high-throughput assay (HTA). Here, we describe the development of a HTA for measuring starch digestibility based on the single-enzyme system [33], which utilizes a 96-sample format for simultaneous starch digestion analysis over a 90 min period. The HTA allows smaller amounts of flour to be analyzed in a standardised 96 well-format using only a thermomixer compared to a cabinet incubator, tube rotator and water bath. These modifications were made to tailor the method for screening large wheat germplasm collections accurately and efficiently. Using standard samples of wheat and maize starch, we validated the assay by comparing starch digestibility profiles to those produced by the single-enzyme system protocol reported in Edwards et al., 2019 [33]. The HTA was then used to screen for the first time processed flour samples of the entire c.Watkins collection [22] as well as elite UK varieties (representing commercial wheat lines for bread, biscuit, and animal feed), which revealed natural phenotypic variation for starch digestibility.

## 2. Materials and Methods

### 2.1. Chemicals

Chemicals used in this study: PercollTM (17-0891-01, GE Healthcare), 4-Hydroxybenzhydrazide (PAHBAH) (5351-23-5), TRIS (77-86-1), EDTA (60-00-4), SDS (151-21-3), DTT (3483-12-3), Phosphate buffered saline (PBS) (P4417-100), Sodium Carbonate (497-19-8), DMSO (67-68-5), maltose (6363-53-7), sodium hydroxide (1310-73-2) and α-amylase (DFP Treated, Type I-A, saline suspension, 647-015-00-4) were purchased from Sigma-Aldrich Company Ltd., Poole, UK.

### 2.2. High-Throughput Starch Digestibility Assay

**Figure 2:**
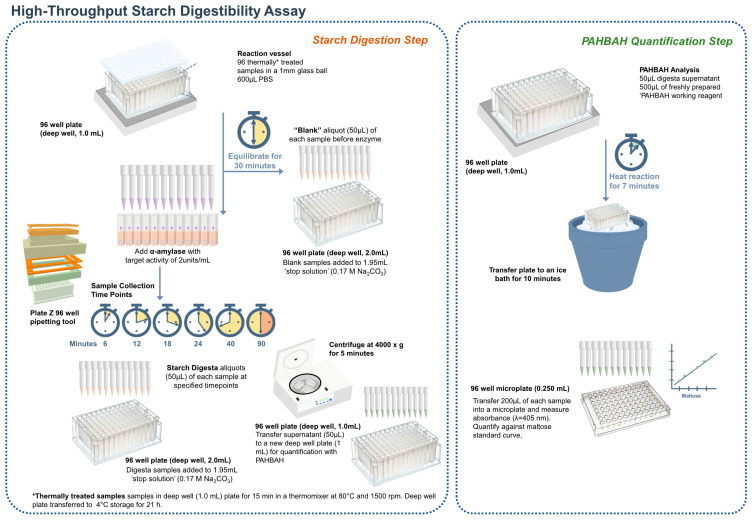
Principle of the high-throughput in vitro starch digestibility assay using a single enzyme system. For amylolysis, a known enzyme-substrate ratio is used, and starch is hydrolysed by porcine pancreatic α-amylase to produce reducing sugars. During amylolysis, aliquots are transferred to a ‘stop solution’ at predetermined time points to inactivate amylase activity. The reducing sugar concentration is quantified using a colorimetric p-hydroxybenzoic acid hydrazide (PAHBAH) assay and maltose standards [36]. The portion of starch digested for each timepoint is calculated based on reducing sugars and is then displayed against time.

A graphical scheme of the HTA assay is presented in Figure 2. Starch digestion assays were carried out on samples that were gelatinized and cooled to accelerate retrogradation following a protocol by Edwards [33], modified for screening a large number of samples. Wholemeal flour samples were weighed (6 mg) and transferred into a deep well plate (96/1000 µL, Eppendorf, Stevenage, UK). Phosphate buffered saline (600 μL, PBS, pH 7.4) was added to each sample with a 1 mm glass ball to improve mixing. The deep well plate was sealed and secured with a Cap-mat (96-well, 7 mm, Round Plug, Silicone/PTFE) and added to a preheated thermal mixer (80 °C) with a 96 SmartBlock™ DWP 1000 n attachment (Thermomixer C, Eppendorf Ltd., Stevenage, UK) for 15 min at 1500 rpm to gelatinize the starch, then cooled at 4 °C for 21 h to accelerate retrogradation. The plate was then briefly spun (100 g for 1 min) to collect condensed liquid on the Cap-mat and placed on the thermal mixer for 30 min at 37 °C, 1600 rpm. Time zero samples (50 μL) were collected using a 12-multichannel pipette and transferred into a 2 mL deep well plate containing 1.95 mL of stop solution (17 mM NaCO_3_). Digestion was started by adding pancreatic α-amylase suspended in PBS targeting 2 U/mL activity into the samples. Enzyme activity was determined by applying the starch digestibility assay on gelatinized potato starch and obtaining the linear rate of maltose release every 3 min (mg/mL). Aliquots (50 μL) were then taken after 6, 12, 18, 24, 40, and 90 min from the onset of digestion and transferred to the stop solution.

The stopped reactions were then centrifuged at 4000× *g* for 5 min to avoid transferring any starch remnants, and 50 μL of the supernatant was transferred to a new deep well plate; 50 μL of maltose standards (5–1000 μM) were also added to the plate. The PAHBAH reducing end assay was used to quantify the reducing ends released [36]. Briefly, 0.5 mL of freshly prepared reagent (2 g of p-hydroxybenzoic acid hydrazide dissolved in 38 mL of 0.5 M HCl and 360 mL of 0.5 M NaOH) was added to each sample. The deep-well plate was held at 100 °C for 7 min and then placed in an ice bath for 10 min. Samples were then transferred to a microplate, and absorbance was measured at 405 nm using a microplate reader (Bio-Rad Benchmark Plus, Waukegan, IL, USA). Reducing sugars were expressed as maltose equivalents, using a standard curve of maltose standards (5–1000 μM) from each sample plate. Starch digestibility (%) was expressed according to the single-enzyme system; each timepoint’s maltose equivalents were corrected by subtracting the baseline maltose (time zero) and then divided by the maltose equivalent of total starch. Four technical replicates were used, each carried out on a different day.

### 2.3. Validation of the High-Throughput Starch Digestibility Assay

Starch digestion profiles from the HTA and the single-enzyme system were compared using standards of purified starch from standard maize, waxy maize, and high-amylose maize (purchased from Merck, formerly Sigma-Aldrich, Darmstadt, Germany), standard wheat starch (purchased from Merck, formerly Sigma-Aldrich, Darmstadt, Germany), and two high-amylose starches: sbeII and ssIIIa, previously characterized, respectively, by Corrado et al. and Fahy et al. [2,21]. Starch samples were aliquoted to make 5.4 mg of starch/mL of PBS, and α-amylase activity was adjusted at 2 U/mL. The thermal treatment procedure was followed as described above for the starch digestibility HTA. Three runs were obtained for both protocols over different days. For each run, six replicates per starch sample were placed randomly in the 96-well plate for the HTA, and two replicates were used for the standard protocol. For each run, starch samples and enzyme solution were prepared in stock and aliquoted for use in both the HTA and single-enzyme system.

### 2.4. Field Trial Design

Grains for the c.Watkins lines were ordered from the Germplasm Resources Unit (John Innes Centre in Norwich, UK) using the publicly accessible SeedStor system https://www.seedstor.ac.uk/, (accessed on 16 November 2022); permission to use the materials for research purposes was obtained. The 118 c.Watkins lines were grown in Autumn 2018 in 1 m^2^ plots (one plot per line, except for the low yield lines) at Church Farm, Norfolk UK (52°37′49.2″ N 1°10′40.2″ E) using standard agronomic practices (Supplemental Figure S1). Based on yield data from previous years, lines with a lower yield performance were grown in duplicate or triplicate (to ensure production of sufficient grains), and grains were pooled for analysis (Supplemental Figure S2). Seeds from elite varieties of bread, biscuit, and animal feed commercial groups of the UK AHDB Recommended List (Cougar, Crusoe, Dickens, Diego, Myriad, Paragon, Santiago, Skyfall) were kindly provided by Brendan Fahy [37] (ahdb.org.uk) (accessed on 16 November 2022). Elite varieties were grown in 2013 in plots (one per genotype) of 1.5 m^2^ at Morley Farm, Norfolk, UK (52°33′15.57″ N 1°10′58.956″ E).

### 2.5. Milling and Sieving

Grains from the c.Watkins and elite varieties were coarsely milled in a cyclone mill fitted with a 0.5 mm screen (UDY Corporation). Milled samples were passed through a 0.3 mm sieve (Endecotts Limited, London, UK) to produce ‘wholemeal’ flour samples, and selected lines were passed through a 0.053 mm sieve to produce ‘sieved’ flour samples. The flour samples were kept in a vacuum desiccator for five days before analysis.

### 2.6. Starch Isolation

Starch was isolated using an adapted method reported in Hawkins, et al. [38]. Wheat flour samples were resuspended with water and filtered through a 100 μm cell strainer (BD Falcon #352360). Samples were then centrifuged at 3000× *g* for 5 min, and the pellets were resuspended in 2 mL of water. The starch suspensions were then overlayed into a Percoll solution (90% *v*/*v*) and centrifuged at 2500× *g* for 15 min to remove cell walls and proteins. The recovered starch pellets were washed with 1 mL buffer (50 mM Tris-HCl, pH 6.8; 10 mM EDTA; 4% SDS; and 10 mM DTT), transferred into a 2 mL tube and left to incubate for 5 min. The starch suspension was then centrifuged at 4000× *g* for 1 min. The pellets were recovered, and the washing procedure was repeated once more. The pellets were then washed three times with 1 mL of water, then once with 100% ethanol. Samples were then kept one day in the fume hood, followed by five days in a desiccator containing silicon dioxide prior to analysis.

### 2.7. Total Starch Assay

Wholemeal flour samples were weighed (~8 mg) and transferred into a deep well plate (96/1000 µL, Eppendorf), each well containing 20 μL of DMSO and a 3 mm glass ball to improve mixing. The plate was mixed for 5 min at 1600 rpm to disperse the samples before adding 500 μL of a thermostable α-amylase to each sample. The thermostable α-amylase was solubilized at 1:30 (*v*/*v*) in 100 mM sodium acetate buffer, pH 5.0 (Total Starch hexokinase kit, AOAC Method 996.1 1; Megazyme, Bray, IE). The deep well plate was sealed and secured with a Cap-mat (96-well, 7 mm, Round Plug, Silicone/PTFE). Samples were heated at 90 °C for 10 min at 1600 rpm using a thermal mixer with a 96 SmartBlock™ DWP 1000 n attachment (Thermomixer C, Eppendorf Ltd., Stevenage, UK). Total starch content was determined using a Total Starch HK kit (Total Starch hexokinase kit, AOAC Method 996.1 1; Megazyme, Bray, IE, Wicklow, Ireland) following manufacturer instructions, except volumes of reagents were scaled down by a factor of 10.

### 2.8. In Depth Analysis of Selected High- and Low-Digestibility Lines

Methods for particle size analysis of flour and starch, size exclusion chromatography, protein content and endogenous α-amylase are available in Supplementary Materials.

### 2.9. Tools

During optimization of the HTA, a low-cost 3D-printed pipetting tool was developed, which allowed for manageable weighing and transferring of samples into 96-sample deep well plates and improved speed and control of pipette aspiration (Plate Z). The PLZ 3D design is available to download and print for free (https://www.hackster.io/386082/high-throughput-pipetting-plate-z-bde2c7) (accessed on 16 November 2022).

### 2.10. Data Analysis

Statistical analyses and graphs were produced using RStudio (R version 4.2.1, Posit Software, Boston, MA, USA). Datasets of the validation of the in vitro starch digestibility HTA were analysed using the packages lme4 (v 1.1-30) and lmerTest (v 3.1-3) for a mixed model fit [39,40,41]. Plots were made using the ggplot2 package (v 3.3.6) [42].

For validation of the HTA, the methods were compared by plotting the estimated starch digestion profiles from the HTA and the single-enzyme system (protocol from Edwards et al., 2019 [33]) and by comparing the estimated starch digested at 90 min. The bias (difference in estimates between methods for the same material) and variation between runs (technical variation) in starch digested at 90 min was estimated using linear mixed models, including the type of starch as a fixed effect and sample batch as a random effect. The variances were estimated using separate models for each method, so that the variability of each method could be compared, while the bias was estimated using a single joint model including all data points, with ‘method’ corresponding to an additional fixed effect.

A linear mixed model including line as a fixed effect and experimental run as a random effect was used for analysis of in vitro starch digestibility of the c.Watkins lines and elite varieties. Marginal means with standard errors (calculated using a pooled standard deviation) are plotted for each line.

The correlation between starch digestibility and total starch was estimated using linear regression. All values reported represent the mean, and the number of replicates and variance metrics are specified in the description of the corresponding figure and Supplementary Datasets.

For complete details of analyses, all data and analysis code is available as Supplementary Materials.

## 3. Results and Discussion

### 3.1. Screening of c.Watkins Landraces and Elite Varieties

#### 3.1.1. In Vitro Starch Digestibility

The aim of this experiment was to determine the extent of natural variation in starch digestibility of the c.Watkins collection and compare it with elite UK varieties representing each commercial group (qualities of bread, biscuit and animal feed).

**Figure 3:**
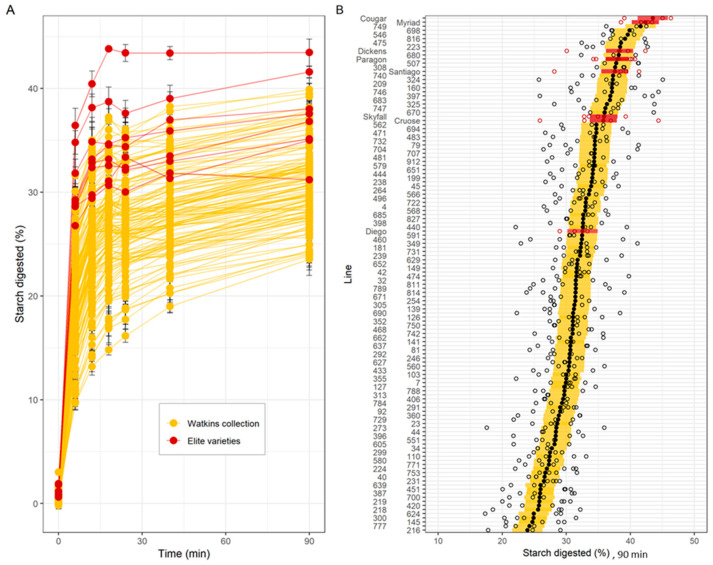
Starch digestibility and total starch of c.Watkins landraces (yellow) and elite varieties (red) of wholemeal flour. (**A**). Starch digested (%) for c.Watkins (yellow) and elite varieties (red). Points and lines represent the mean and standard error from between 3 and 6 technical replicates per line. (**B**). Starch digested (%) at 90 min. Individual data points from technical replicates are shown in white dots, and marginal mean values from a mixed effects model with line as a fixed effect and experimental run as a random effect are shown in black (c.Watkins) and red dots (elite). The standard error estimated from the model is displayed as yellow (c.Watkins) and red (elite) bars. Values represent mean ± SE of *n* ≥ 3 replicates.

Results of the HTA revealed a wide range of variation in starch digestibility among c.Watkins landraces; starch digestibility profiles formed a gradient of low- to high- digestibility rather than two distinct groups, as expected from complex traits (Figure 3A shows the individual trajectories of starch digested over time, Figure 3B compares the starch digested for each line at 90 min). For the c.Watkins lines, the levels of starch digested at 90 min ranged between 9.7–31.6% (at 6 min), 13.2–35% (at 12 min), 14.8–37.2% (at 18 min), 16.2–36.1% (at 24 min), 19–37.8% (at 40 min), and 23.5–39.9% (Figure 3B). The residual variance from the mixed effect model, measuring the variability between technical replicates from the same sample in the same run, was 3.7 percentage points. There was no statistical evidence for a relationship between the plot location in the field and the starch digestibility at 90 min, as no trend was observed between field rows and columns (Supplemental Figure S3).

Elite UK varieties showed less variation and greater starch digestibility compared to most c.Watkins lines, although a direct comparison is confounded by the different growing conditions between the two groups (Figure 3A). For example, the levels of starch digested for the elite varieties ranged between 26.8–36.4% (at 6 min), 29.4–40.4% (at 12 min), 30.6–43.8% (at 18 min), 30–43.4% (at 24 min), 31.3–43.4% (at 40 min) and 31.2–43.5% (at 90 min) (Figure 3B).

Our results demonstrate that natural phenotypic variation for starch digestibility exists in the c.Watkins collection (representing the genetic diversity of the entire Watkins collection, 826 lines) as well as in elite varieties representing commercial groups of bread, biscuit and animal feed groups, and so there is potential for identifying underlying genetic diversity. The variation observed among the c.Watkins lines was greater than among the elite varieties screened, which is consistent with the higher levels of genetic diversity previously reported for the c.Watkins collection compared to modern European bread wheat varieties, as well as increased phenotypic variation for agronomically desirable traits, such as stripe, leaf, and stem rust resistance, as well as grain surface area and grain width [22,23,24,25,26]. It is important to note that a limitation of our study was that the grain samples of c.Watkins lines and elite varieties were not produced in the same field trial, and each a single plot per line, so it is possible that environmental conditions between and within fields could influence the average starch digestibility levels and the variation observed; this will be important to consider in future trials. With the availability of structured germplasm panels (nested association mapping populations) and genotypic data [43], there is now potential for use of the HTA to investigate the genetic factors underlying the variation in starch digestibility observed through approaches like QTL analysis which will aid in identification of underlying candidate genes and development of tools for marker assisted-selection. Furthermore, once lines are identified, digestion of starch and other nutrients in wheat foods can be explored using more extensive in vitro models of digestion in the upper gastrointestinal track such as the standardized INFOGEST protocol, based on an international consensus by COST INFOGEST network [44].

#### 3.1.2. Total Starch

Total starch (TS) content of wholemeal flour varied significantly for c.Watkins landraces (43 ± 3.3 g/100 flour to 61 ± 2.4 g/100 flour, mean ± SE, *p* ≤ 0.001), and the elite varieties (46 ± 3.6 g/100 flour to 61 ± 0.9 g/100 flour, mean ± SE, *p* ≤ 0.05). The elite variety Diego had the highest TS content (61 ± 0.9 g/100 flour, mean ± SE), and the c.Watkins line 651 had the lowest (43 ± 3.3 g/100 flour, mean ± SE). Most of the samples had a TS content between 47 to 57 g/100 flour. Linear regression analysis showed that total starch content only weakly correlated (R^2^ = 0.0108) with starch digested at 90 min which suggests that the differences in total starch content do not explain the variation observed for starch digestibility (Supplemental Figure S4).

#### 3.1.3. Analysis of Low- and High-Digestibility Lines

To gain further insight into factors influencing the difference in starch digestibility a subset of c.Watkins lines and elite varieties were selected, based on their starch digestibility profiles (high- vs. low-digestibility) to measure starch digestibility in sieved flour (to obtain smaller particle size fractions) and purified starch.

Three low-digestibility lines: 777 (WATDE0111), 216 (WATDE0025), and 639 (WATDE0083); and five high-digestibility lines: including two c.Watkins landraces 816 (WATDE0117), 308 (WATDE0042) and the three UK elite varieties Myriad, Dickens, and Paragon were selected for further analysis. The selection was based on the starch digestibility HTA results, specifically the ranking (low to high), to represent the variation observed in the screen.

**Figure 4:**
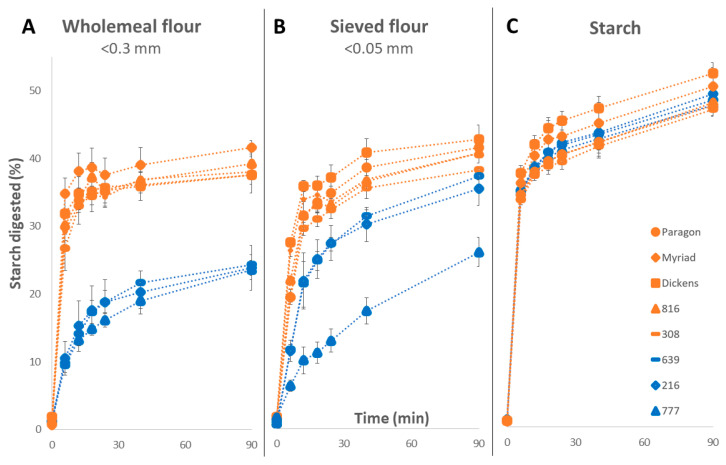
Starch digestibility of selected low- and high-digestibility lines measured in wholemeal flour (**A**), sieved flour (**B**) and purified starch (**C**) using the HTA. Samples in blue represent the low-digestibility lines, and samples in orange represent the high-digestibility lines. Values represent mean ± SE of *n* = 3 replicates.

Results presented in Figure 4 show that starch digestibility profiles differed considerably for wholemeal flour, sieved flour, and purified starch, suggesting that other flour components besides starch likely may contribute to the variation in starch digestibility observed. For wholemeal flour (<0.3 mm), starch digestibility profiles of selected ‘low-’and ‘high-digestibility’ lines grouped separately where the high-digestibility samples varied between 37.6–41.6% and low-digestibility samples varied between 23.5–24.3% (at 90 min) (Figure 4A). For sieved flour (<0.05 mm), the digestibility of two low digestibility-lines (216 and 639) increased significantly (*p* < 0.05) compared to the corresponding wholemeal samples, and while the third low-line (777) remained low (Figure 4B). Purified starch samples showed a greater extent of starch digested at 90 min compared to wholemeal and sieved flour fractions (Figure 4C). Moreover, there were no differences between the high- and low-digestibility groups in purified starch samples. For example, high-digestibility lines differed between 46.9–52.3% and low-digestibility lines varied between 47.6–49.2% (at 90 min).

Finally, these selected lines were also analyzed for starch structural properties (starch chain-length distribution and starch granule size distribution) and flour properties (flour particle size, endogenous α-amylase and protein content). These results are described below. In summary, while differences between the lines themselves were evident for many properties, none of these factors significantly correlated with starch digestibility in this sample (Supplemental Table S1 and Supplemental Figures S5–S7).

Starch molecular structure and composition have been shown to influence its digestibility; thus, starch fine molecular structure and starch granule size-distribution was examined in the selected low- and high-digestibility lines. Starch chain-length distributions showed significant differences between lines in the proportion of long and short chains of amylopectin (AP) and amylose (AM) (*p* < 0.001) (Supplemental Figure S5 and Table S1). However, the overall AM:AP ratio was not significant. Starch granule size distribution of purified starch using a Coulter counter revealed significant variation among starch granule diameter (*p* < 0.001), whereas minor differences were observed for the overall volume of A and B granules (Supplemental Table S1). Low-digestibility lines showed the greatest variation in B-granule diameter, with 6 ± 0.1 μm SE for line 777 to 8.4 ± 0.2 μm SE for line 216 (40% greater). The diameter of B-granules in high-digestibility lines varied less; 816 had the highest (6.9 ± 0.4 μm SE), and 308 had the lowest (6.2 ± 0.1 μm SE). In general, similar trends were also observed for A-granule diameter, except that low-digestibility line 216 had significantly larger A-granules. The diameter of A-granules from low digestibility lines ranged from 18.2 ± 0.2 μm SE (lines 777 and 639) to 20.1 ± 0.2 μm SE (line 216). There was also significant variation in A-granule diameter among high-digestibility lines; 308 had the smallest (17.7 ± 0.04 μm SE) and Paragon the largest (20.1, ± 0.2 μm SE). Overall, we observed differences in chain-length distribution profiles and starch granule size distribution of the selected lines but the there was no correlation of either factor to starch digestibility of the wholemeal flour.

Starch digestibility has also been shown to be affected by the particle size of wheat flour [17]. Particle size analysis of wholemeal flour from selected low- and high-digestibility lines showed no major variation (Supplemental Figure S6). There was a statistically significant difference (*p* < 0.003) in the particle size of wholemeal flour; line 639 had more particles in the range of 14–20 μm whereas Paragon showed a slightly smaller number of particles in that range. Micrographs of wholemeal flour (produced using scanning electron microscopy) showed aggregate formations ≥ 120μm in all lines apart from the low-digestibility line 639, which only had particles smaller than 120μm (Supplemental Figure S7). This data suggests that the milling efficiency was similar in all lines apart from 639.

Elevated amounts of endogenous α-amylase in cereals could trigger early starch amylolysis in the endosperm and thus significantly affect starch digestibility [45,46]. The activity of endogenous α-amylase in low- and high-digestibility lines differed significantly (*p* < 0.05), (Supplemental Table S1), however values were within a normal range (<0.2 Ceralpha Units/g) according to prior studies (McCleary et al., 2002, Derkx and Mares, 2020). The activity of endogenous α-amylase in low-digestibility lines ranged from 0.06 ± 0.01 to 0.15 ± 0.01 Ceralpha Units/g of flour, mean ± SD, with the lowest in line 639 and the highest line 216. High-digestibility lines varied from 0.05 ± 0.01 to 0.13 ± 0.01 Ceralpha Units/g of flour, mean ± SD, Paragon being the lowest and line 308 the highest.

Grain hardness can impact starch digestibility and is mainly affected by the protein content and composition in the endosperm [47]. Thus, protein content of wholemeal flour was analysed in the selected lines. Results showed that protein content varied significantly (*p* < 0.001) (Supplemental Table S1); low-digestibility lines ranged from 14.2–18.4 g/100 flour, mean and high-digestibility lines ranged from 10.6–17.4 g/100 g flour, mean.

The different digestibility profiles observed across purification steps (wholemeal flour to sieved flour to purified starch) suggest that multiple mechanisms in the selected lines could be affecting starch digestibility and that different factors in each line could have a distinct effect. This is consistent with prior studies which have shown effects of flour characteristics and starch structure on starch digestibility in cooked wheat samples including particle size of wholemeal flour, protein content, and chain-length distribution of starch polymers [31,48,49]. An important limitation of this aspect of the study was the small number of lines selected for this analysis severely limiting the power to detect correlations of phenotypes between lines; prior work by Wang et al. [29] identified starch properties influencing starch digestibility in a screen of 224 wheat starches. Nevertheless, our study suggests that a combination and interaction of many factors are required to achieve low starch digestibility profiles in flour, so using a starch digestibility HTA for screening or selection approaches may be more efficient and informative than selection based solely on underlying factors such as starch molecular structure.

### 3.2. Establishment and Validation of a High-Throughput Starch Digestibility Assay

The aim of this experiment was to validate and assess the reliability of the 96-sample format starch digestibility assay using established starch standards of maize and wheat by comparing the HTA with the single-enzyme system protocol presented in Edwards et al., 2019 [33].

**Figure 5:**
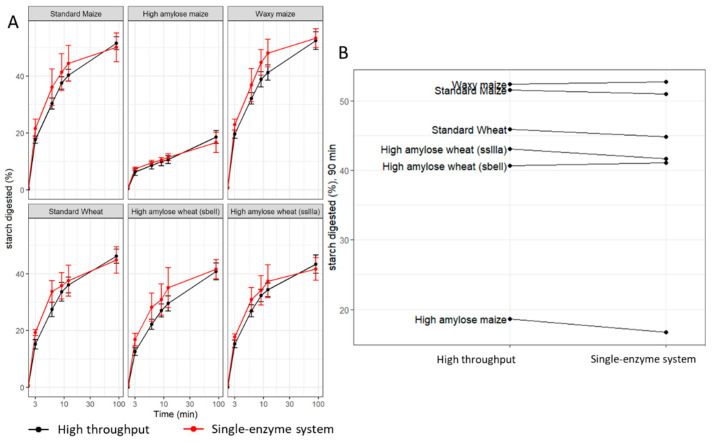
Comparison of the HTA to the single-enzyme system protocol reported in Edwards et al., 2019. (**A**) Digestibility profiles of purified maize and wheat starch produced by the HTA (black) and the single-enzyme system (red). (**B**) Starch digested (%) at 90 min, ranking comparison of the HTA (left) to the single-enzyme system (right). Values represent the mean ± SD of *n* = 18 replicates for the HTA and *n* = 6 for the single-enzyme system.

Digestion profiles of maize and wheat starch standards were comparable to profiles generated using the single-enzyme system protocol in Edwards et al., 2019 [33] (Figure 5A). For example, the difference in the average estimates at 90 min (Figure 5B) from the two methods was −0.72 percentage points (not statistically significant), and the technical variation observed at 90 min within the runs was 2.1 percentage points in the HTA and 2.2 percentage points in the single-enzyme system protocol, suggesting that the HTA is not biased and is no less reliable.

The HTA (detailed in the Section 2) was scaled from a 6-tube format to a 96-well plate format which required optimizations for maintaining even heat distribution during starch digestions and PAHBAH assays, for the mixing ability of samples, and for recovering adequate sample volumes for analysis. We also developed new tools to allow faster sample handling for sample preparation and pipetting (Plate Z, available free to download from hackster.io, (accessed on 16 November 2022). A graphical scheme is presented in the Supplemental Materials highlighting the changes made to develop the HTA (Supplementary Figure S8).

There were no significant differences in the percent of starch digested at all the time points measured between the single-enzyme system protocol and the HTA. Thus, the HTA provided comparisons between samples that were accurate and reproducible, and the reliability of the assay was sufficient for use as a screening tool to aid in the selection of low- and high-digestibility samples.

The variance using wholemeal flour (in the c.Watkins screen, Section 3.1.1) was higher than the variance using starch standards in the validation experiment which could be due to the higher variability in wholemeal flour composition compared to purified starch samples. The number of technical replicates needed to estimate starch digestibility to a given precision can be calculated from residual variance.

Recent studies have reported improved methods to increase the throughput of starch digestibility assays. Wang et al. [29] developed a 96-sample format assay to screen starch from a wheat MAGIC population and used a multifactorial analysis to identify the most influential factors for starch digestibility (starch granule size distribution, amylopectin chain length distribution, amylose content and endogenous α-amylase activity). Other studies have also aimed to increase the throughput of starch characterization techniques however, the starch properties analyzed may or may not have a direct impact on starch digestibility [50,51]. It is important to consider that other components of wheat flour and factors like processing are likely to have a major impact on starch digestibility thus, determining starch digestibility on processed flour samples may better represent what people may consume in wheat-based foods [29,30,33,53]. Some progress has been made for other crops like rice; Toutounji et al. [52] developed a 15-sample format assay for screening starch digestibility of cooked rice grains with potential to allow analysis of 60 samples per day (if 15 samples are prepared every two hours). In this method, samples were handled individually with a single pipette, which was a limiting factor for the number of samples that could be processed simultaneously. Furthermore, the assay was performed using a set of 8 samples and this assay still needs to be validated on a larger sample set. The HTA assay described here addresses some of these key limitations presented by prior assays namely the type of sample analyzed (hydrothermally processed flour) and sample size (96-sample format). For future work, it will be important to consider improving the efficiency of upstream steps, including milling, sieving, and weighing samples which present additional bottlenecks.

## 4. Conclusions

In this study we identified wide variation in starch digestibility among the c.Watkins lines which could be used to recover valuable alleles that were lost during modern breeding with the aim of improving wheat nutritional quality. Starch digestibility profiles of sieved flour and purified starch from selected lines suggested that the differences observed in starch digestibility are likely due to multiple factors in the flour matrix and are not limited to starch structural properties. Previously, strategies to reduce starch digestibility in wheat have mainly focused on reverse genetic approaches, but with new tools like the HTA there is potential to discover other useful sources of variation to support development of wheat varieties with improved health benefits. Furthermore, future work to determine starch digestibility in white flour samples will be useful for identifying sources of reduced starch digestibility for applications in foods made with refined flour.

## Data Availability

Data sets of starch digestibility and total starch and all statistical analysis code are included as Supplementary Materials.

## Supplementary Materials

The following supporting information can be downloaded at: https://www.mdpi.com/article/10.3390/foods12020266/s1, Supplemental.docx, data sets of starch digestibility and total starch, and all statistical analysis code. Additional references cited in Supplementary Materials: [53,54].
