# Supplementary material for: Mining the A.E. Watkins Wheat Landrace Collection for Variation in Starch Digestibility Using a New High-Throughput Assay
Source: Foods. 2023 Jan 6;12(2):266. doi: 10.3390/foods12020266 (PMC9858048; doi:10.3390/foods12020266)
Supplement: Supplementary file 1 [file foods-12-00266-s001.zip › foods-2070158-supplementary.pdf]

	698	349
	694	324
	694	313
	690	Soison
	690	313
	685	308
	Soison	305
	685	300
	683	299
	680	292
	671	291
	670	352
	662	273
	652	264
	639	254
	637	325
	629	246
	627	239
	627	238
	Soison	Soison
912	624	231
827	605	224
827	591	Soison
827	591	223
816	580	219
816	Soison	218
814	579	216
811	568	Soison
Soison	568	209
811	566	209
789	562	Soison
788	560	199
784	551	199
777	546	181
771	507	160
753	496	149
750	483	145
749	481	141
747	475	139
Soison	Soison	Soison
746	474	127
742	471	Soison
742	471	126
740	468	110
651	Soison	103
732	468	92
731	460	81
Soison	451	79
731	444	Soison
729	440	45
729	433	44
722	420	42
707	406	40
705	398	34
705	397	34
704	396	32
700	387	23
700	360	7
698	355	4
Soison	Soison	Soison

Figure S1. *Field trial (1 m^2 plots at Church Farm, Norfolk UK (52°37'49.2"N 1°10'40.2"E)) of c.Watkins. Numbers represent the lines, and Soison was used as a standard for the field trial validation.*

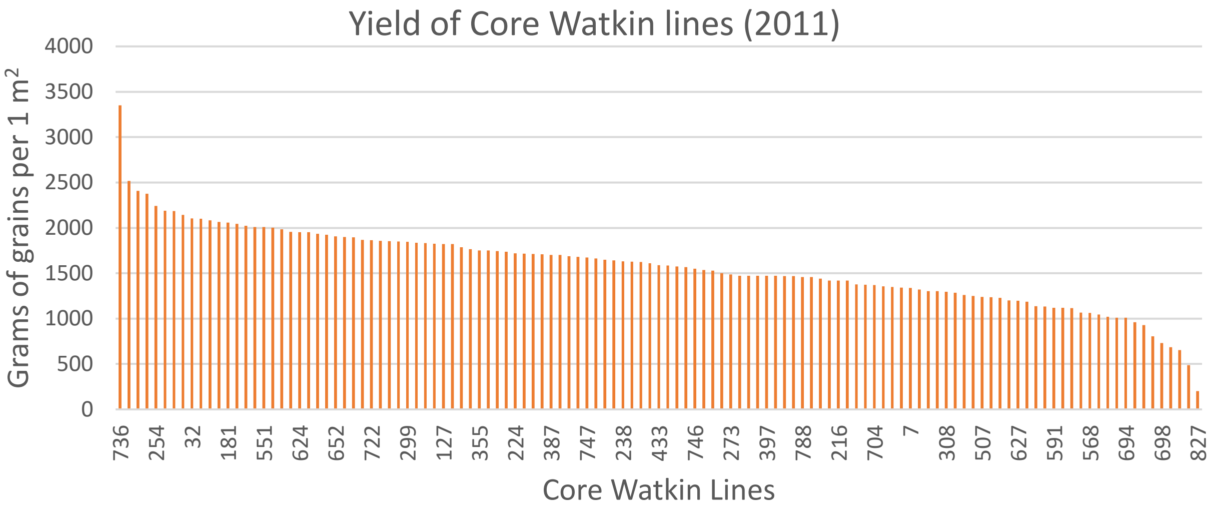

Figure S2. *Core Watkins' collection yield (2011). Provided by Germplasm resources unit.*

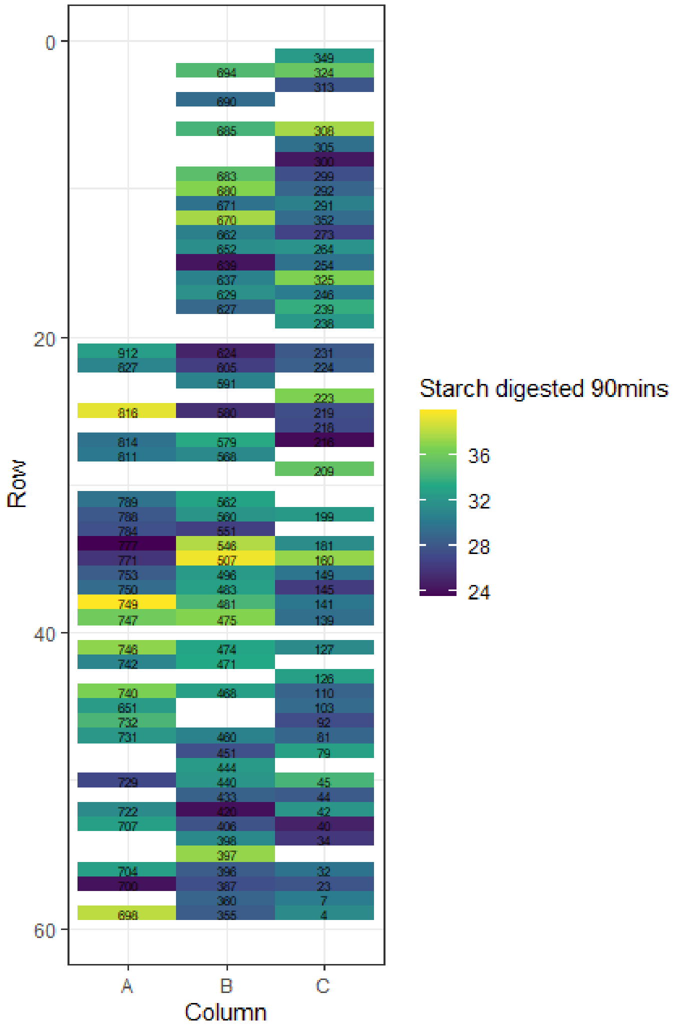

Figure S3. *Field design of c.Watkins collection and starch digested (%) at 90 min.*

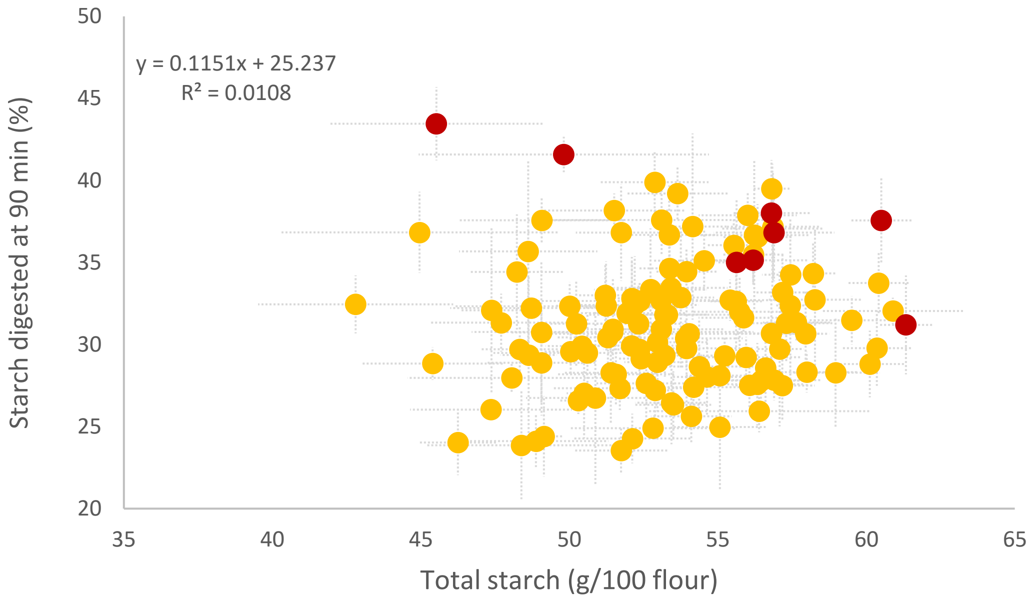

Figure S4. *Scatter plot of starch digestibility (%) and total starch content (g/100 flour) of c.Watkins landraces (yellow) and elite varieties (red). Error bars represent ± (SE).*

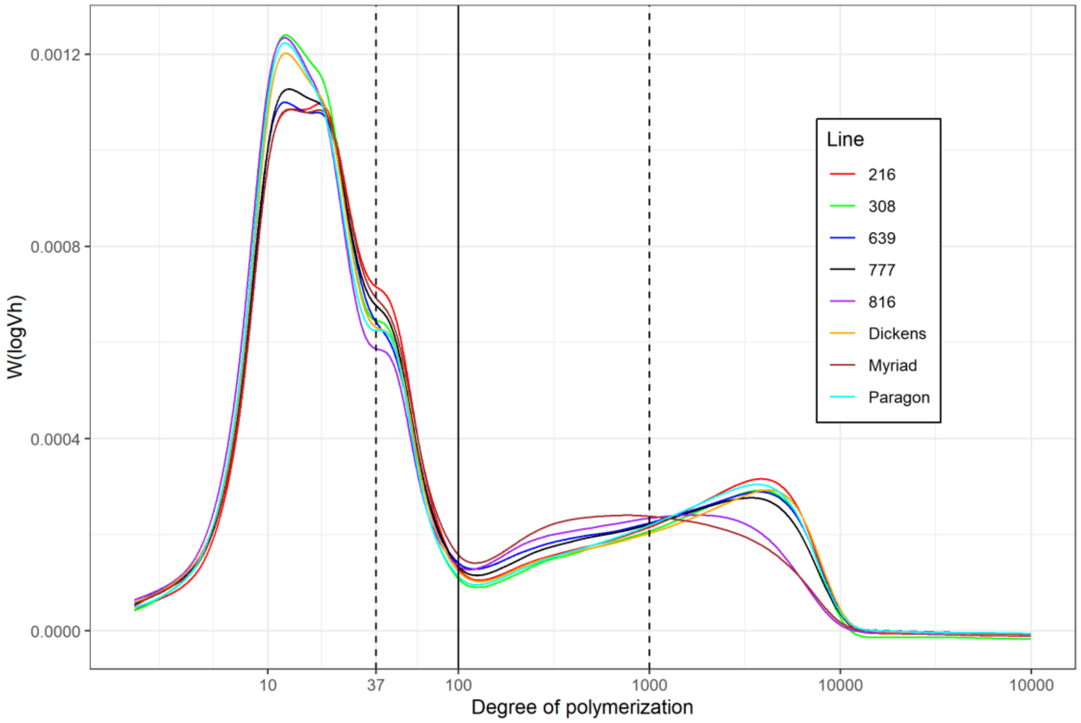

Figure S5. *Starch chain-length distribution for selected wheat lines (216, 308, 639, 777, 816, Dickens, Myriad and Paragon). The Y-axis is expressed as wLogVh; the X-axis is the degree of polymerization. The curves were normalised such that the sum of each was 1 over the plotted range. Each curve represents the mean of three technical replicates (except line 308 which had 2 technical replicates).*

Table S1. *Protein content of wholemeal flour was measured on a fresh weight basis. Coulter counter analysis for granule size distribution described as the relative volume of B-type granules (%), B-type granule diameter (μm) and A-type granule diameter (μm). Chain-length distribution was determined by SEC: amylopectin (AP) long to short chains (ratio of 37<x<100 to <37 DP), AM (amylose) long/ short chains (ratio of and >1000 to >100-1000 DP), AM/AP (ratio of >100 to <100). Endogenous α-amylase was determined as Ceralpha Units/g of flour. Values represent mean ± SE. p-values are for comparisons made between the selected lines using ANOVA.*

	**Lines**	**Protein content (g/100 flour)**	**B granule (%)**	**B-type granule diameter (μm)**	**A-type granule diameter (μm)**	**AP long/short chains**	**AM long/short chains**	**AM/AP ratio**	**Endogenous α-amylase Ceralpha Units/g flour**
p-value		p < 0.001	ns	p < 0.01	p < 0.001	p < 0.001	p < 0.005	ns	p < 0.05
Low Digestibility	639	15.1 (0.17)	37.1 (1.9)	6.4 (0.2)	18.2 (0.1)	0.23 (0.01)	0.94 (0.06)	0.32 (0.01)	0.06 (0.01)
	777	14.2 (0.16)	34 (2)	6 (0.1)	18.2 (0.2)	0.24 (0.01)	0.93 (0.05)	0.30	0.07 (0.01)
	216	18.4 (0.05)	28.6 (1.6)	8.4 (0.2)	20.1 (0.2)	0.26	1.10 (0.04)	0.29 (0.01)	0.15 (0.01)
High Digestibility	308	14.9 (0.18)	35.3 (1.7)	6.2 (0.1)	17.7	0.21 (0.01)	1.06 (0.06)	0.26 (0.02)	0.13 (0.01)
	Myriad	11.1 (0.22)	34.2 (2.4)	6.7 (0.1)	19.2 (0.5)	0.25	0.84 (0.13)	0.29 (0.02)	0.06 (0.01)
	816	17.4 (0.19)	34.8 (0.7)	6.9 (0.4)	19 (0.5)	0.20 (0.01)	0.65 (0.05)	0.27 (0.03)	0.09 (0.01)
	Paragon	13.5 (0.24)	30.9 (1)	6.6 (0.1)	20.1 (0.2)	0.21	1.16 (0.14)	0.29 (0.01)	0.05 (0.01)
	Dickens	10.6 (0.15)	29 (3.3)	6.3 (0.2)	19.2 (0.3)	0.22	1.09 (0.04)	0.28	0.05 (0.01)

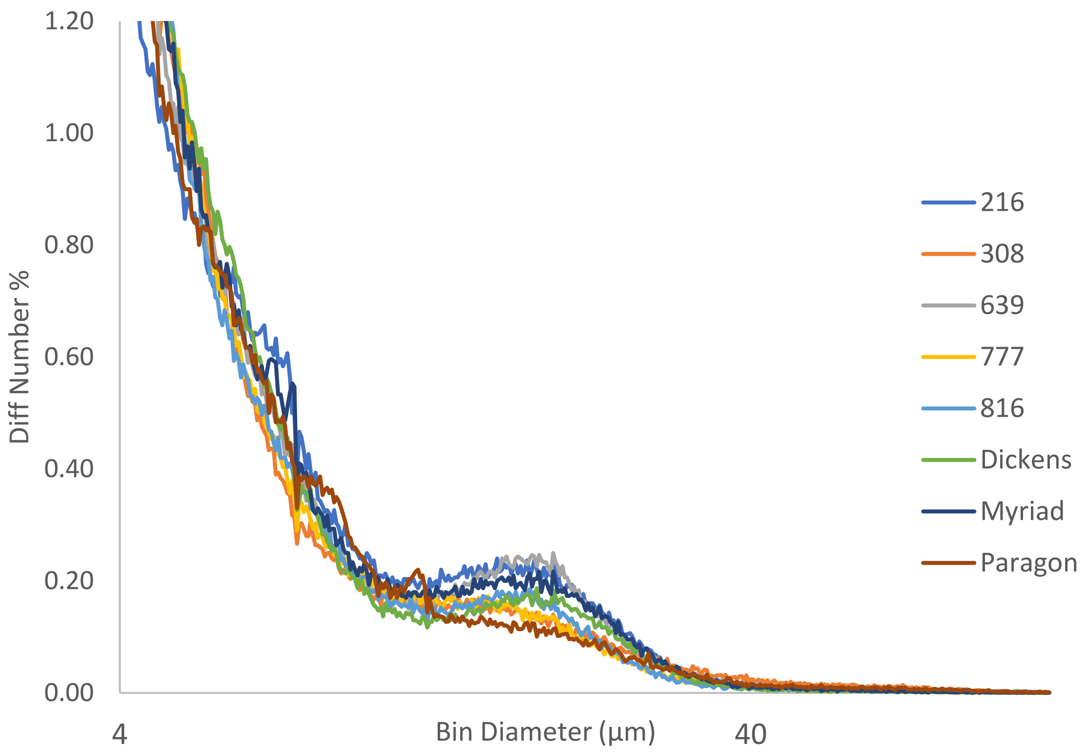

Figure S6. *Differential Particle Size Distribution of wholemeal flour particles by Coulter counter analysis, data is shown on a log scale in the x-axis.*

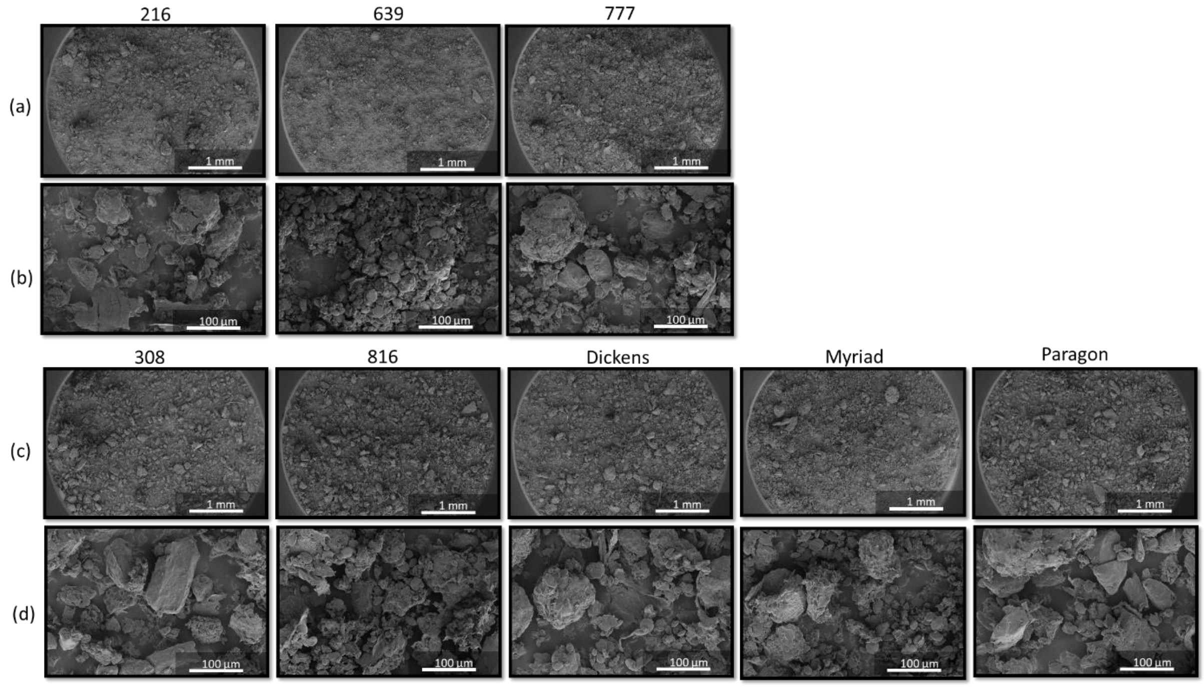

Figure S7. *SEM micrographs of wholemeal flour for low- and high-digestibility lines.* ***(a)*** *and* ***(b)*** *are from of low digestibility lines and* ***(c)*** *and* ***(d)*** *are from high digestibility lines.*

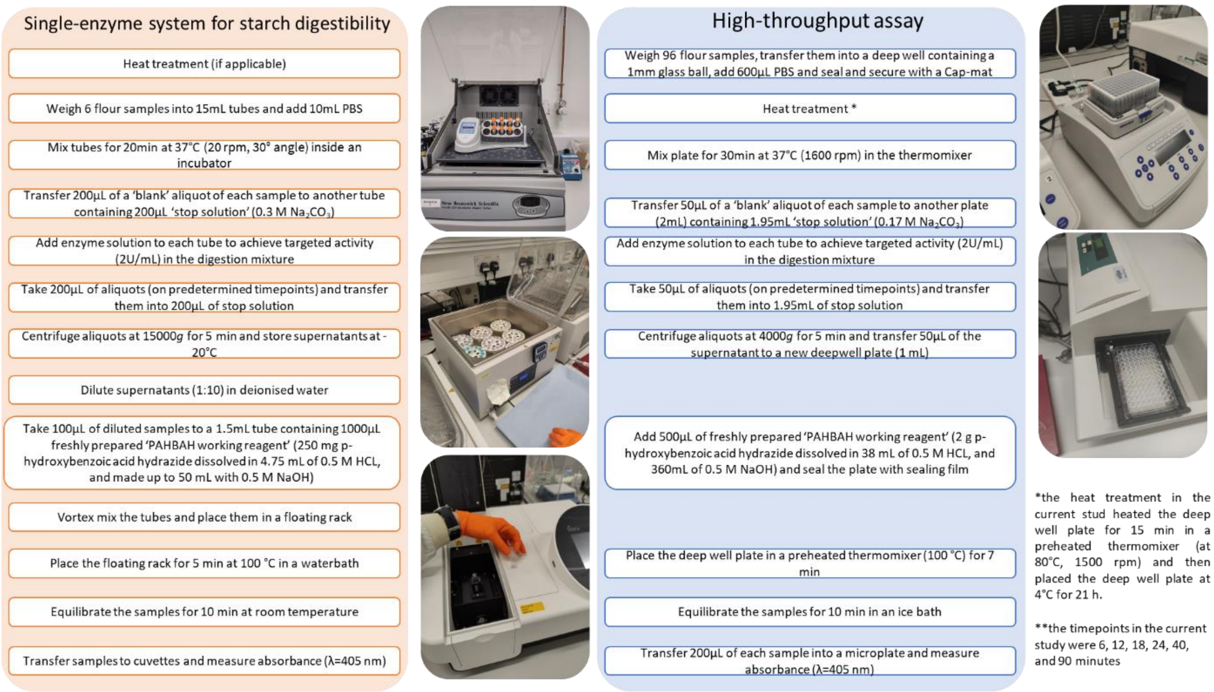

Figure S8. Comparison between the single-enzyme system (protocol from Edwards et al. 2019 [33] for starch digestibility and HTA. *the heat treatment in the current stud heated the deep well plate for 15 min in a preheated thermomixer (at 80°C, 1500 rpm) and then placed the deep well plate at 4°C for 21 h. **the timepoints in the current study were 6, 12, 18, 24, 40, and 90 minutes.

Supplemental methods

Size Exclusion Chromatography

Size exclusion chromatography was used to determine chain-length distribution of debranched starch for select samples. A Waters Alliance e2695 HPLC (Milford, US) equipped with a refractive index detector (RI), autosampler (40°C), and column heater (90°C) was used for peak resolution by size exclusion. An isocratic mobile phase of DMSO with 0.5% LiBr (w/w) at 0.500 mL/min flow rate was used. The stationary phase was a guard column (8x50mm, GRAM; Polymer Standard Service, Mainz, DE) followed by two analytical columns in series (10um; 300 Å followed by 30 Å; 8x300mm, GRAM; Polymer Standard Service, Mainz, DE). Total run time was 65 minutes and injection volume for each sample was 50 µL. Calibration curves were generated using pullulan standards (PSS-pulkit, Polymer Standard Service, Mainz, DE) with peak molecular weights ranging from 342 to 708,000 Da and correlation coefficients of R^2 = 0.9993 ± 0.0005. Purified starch was solubilized and debranched enzymatically using methods adapted from Perez-Moral*, et al.* [54] Debranched starch samples were solubilized at a concentration of 8 mg/mL and standards at 2 mg/mL with DMSO containing 0.5% LiBr (w/w). All samples and standards were vortexed and stored at 80°C overnight prior to analysis. The relationship between elution volume and hydrodynamic radius (Vh) for the linear glucans was determined using calibration curves described by[55] The debranched starch samples RI elution profiles were converted to SEC weight distributions as described in detail by Perez-Moral*, et al.* [54].
For the chain-length distribution analysis, both amylopectin (AP) and amylose (AM) chains were divided into short- and long-chain fractions based on their degree of polymerization (DP) (<37 DP and >37-100 DP for AP; >100-1000 and >1000 DP for AM), then the area under the curve was calculated for each line. To compare the samples, ratios of long/short AP, long/short AM and AM/AP were used.

Protein Content

The protein content of wholemeal flour was measured using the AACC 46-30 Crude protein – combustion method [56]on a CE440 Elemental Analyser (Exeter Analytical, https://www.exeteranalytical.co.uk/). Protein (%) was calculated by multiplying obtained Nitrogen by a factor of 5.7.

Scanning electron microscopy

Wholemeal flour was brushed onto a carbon tab and fixed to a SEM pin stub. Samples were visualised using a Nova NanoSEM 450 (FEI) scanning electron microscope.

Endogenous α-amylase

Endogenous α-amylase levels present in wholemeal flour samples were determined using the Ceralpha assay procedure following manufacturing instructions (α-Amylase Assay Kit (Ceralpha Method), AOAC Method 2002.01; Megazyme, Bray, IE).

Particle size analysis of flour and starch

Particle size analysis of wholemeal wheat flour and purified starch granules was carried out using a Multisizer 4e coulter counter (Beckman Coulter, Indianapolis, US). Samples (5 mg of flour or 10 mg of purified starch) were suspended in 1 mL of deionized water and mixed for 15 min on a rotating wheel. Suspensions were filtered through a sieve (200 µm nylon mesh for the flour and 70 µm nylon mesh for the starch) into 100 mL of Isoton II electrolyte solution (Beckman Coulter) before the analysis. The aperture tube used was 200 µm for wholemeal flour and 70 µm for the purified starch. A minimum of 100,000 particles were quantified. For starch samples, the mean diameter of A- and B-type granules and relative volumes were calculated according to[21]. All measurements were conducted with logarithmic bin spacing but are presented on a linear x-axis for clarity.
